# Enabling “lithium-free” manufacturing of pure lithium metal solid-state batteries through in situ plating

**DOI:** 10.1038/s41467-020-19004-4

**Published:** 2020-10-15

**Authors:** Michael J. Wang, Eric Carmona, Arushi Gupta, Paul Albertus, Jeff Sakamoto

**Affiliations:** 1 Department of Materials Science and Engineering, University of Michigan, Ann Arbor, MI 48109 USA; 2 Department of Chemical and Biomolecular Engineering, University of Maryland, College Park, MD 20742 USA; 3 Department of Macromolecular Science and Engineering, University of Michigan, Ann Arbor, MI 48109 USA; 4 Department of Mechanical Engineering, University of Michigan, Ann Arbor, MI 48109 USA

**Keywords:** Batteries, Batteries, Nanoscale materials

## Abstract

The coupling of solid-state electrolytes with a Li-metal anode and state-of-the-art (SOA) cathode materials is a promising path to develop inherently safe batteries with high energy density (>1000 Wh L^−1^). However, integrating metallic Li with solid-electrolytes using scalable processes is not only challenging, but also adds extraneous volume since SOA cathodes are fully lithiated. Here we show the potential for “Li-free” battery manufacturing using the Li_7_La_3_Zr_2_O_12_ (LLZO) electrolyte. We demonstrate that Li-metal anodes >20 μm can be electroplated onto a current collector in situ without LLZO degradation and we propose a model to relate electrochemical and nucleation behavior. A full cell consisting of in situ formed Li, LLZO, and NCA is demonstrated, which exhibits stable cycling over 50 cycles with high Coulombic efficiencies. These findings demonstrate the viability of “Li-free” configurations using LLZO which may guide the design and manufacturing of high energy density solid-state batteries.

## Abstract (web summary)

While the impetus to develop lithium metal solid-state batteries is clear, identifying a practical manufacturing process is challenging. Herewith, authors study the underlying mechanisms controlling in-situ anode formation that could enable viable lithium-free manufacturing.

## Introduction

Owing to the combination of high energy density and safety, solid-state batteries are a promising candidate to enable the widespread adoption of electric vehicles. Along with the replacement of the flammable liquid electrolyte, solid electrolytes may enable the replacement of graphite anodes with metallic Li, which allows for a dramatic (40–50%) increase in energy density^1–5^. Although advanced cathode chemistries would undoubtedly further improve the theoretical energy densities, it is believed that state-of-the-art cathodes (e.g., NMC, NCA, LFP) are currently the most viable chemistries to achieve energy densities >1000 Wh L^−1^, cycle life > 1000 cycles with ≤80% capacity fade, and current cost < $100 kWh^−1^ targets^6,7^. However, given that current state-of-the-art cathodes are typically manufactured in the fully lithiated state, any pre-deposited Li metal will add extraneous volume.

On the laboratory scale, Li-metal solid-state cells are commonly constructed using thick (>200 μm) Li foils, although Li foils down to ~20 μm have been made. However, given the difficulty and cost of handling, free-standing Li foils may not be viable^8^. In addition to manufacturing, integration of the Li-metal anode with a solid-electrolyte with relevant thickness, low interfacial resistance, high chemical purity, and using scalable processes still remains a major challenge. For these reasons, in both liquid- and solid-state Li-metal batteries, there is a growing interest in “Li-free” (or anode-free) manufacturing^9–12^, in which the battery is fabricated in the discharged state, with a bare current collector (CC) replacing the conventional anode. The Li-metal anode is then formed electrochemically on the first charge cycle by electroplating Li contained within the cathode. In liquid-based batteries, this concept has been demonstrated, but it’s feasibility is limited by the high reactivity of Li with traditional liquid electrolytes, leading to low cycling efficiency^9,10,12–14^.

Lithium phosphorus oxynitride (LiPON) is one of the few stable solid-electrolyte materials that demonstrate the ability to resist Li filament propagation, thereby enabling the fabrication of “Li-free” batteries^15^. Indeed, the pioneering development of thin-film LiPON technology demonstrated the feasibility of Li-metal solid-state secondary batteries; however, current approaches are pursuing bulk-scale manufacturing approaches to achieve cost parity with large format technologies such as Li-ion. One of the most promising solid-state electrolytes is Li_7_La_3_Zr_2_O_12_ (LLZO), which has high ionic conductivity and excellent stability against Li metal. However, unlike most LiPON systems, under certain conditions LLZO is susceptible to Li filament propagation and subsequent short-circuiting^16–18^. Recent studies have investigated the Li nucleation behavior at the interface of sputtered CCs and LiPON or LLZO electrolytes, which allows for visualization of the plating process^19–22^. It was commonly observed that Li plating causes fracture of the CC, leading to “dead Li” formation, low Coulombic efficiencies, and even short-circuiting in the case of LLZO. Thus, although “Li-free” manufacturing has been demonstrated in solid-state systems, the inability to plate significant capacities of Li without either electrolyte or CC fracture severely limits the cycle life of the cell. These issues combined with the limited approaches for large-scale LiPON manufacturing have prevented widespread adoption of “Li-free” solid-state batteries. In addition, it has been hypothesized that the interposition of a Ag-C (∼10 μm) layer can reduce nucleation energy to enable Li-metal plating using Li from an NMC cathode. While reduction in nucleation energy was not quantified, Li plating into and beneath the Ag-C interlayer was clearly demonstrated^23^. However, in principle, so long as physical and electrical contact can be maintained throughout the formation process and subsequent battery operation, the combination of “Li-free” architectures and a stable solid electrolyte is a rational strategy toward practical manufacturing of high-efficiency Li-metal secondary batteries. Given the myriad of complex physical phenomena at play, a deeper understanding of the mechanics and electrochemistry is necessary to assess the feasibility of “Li-free” manufacturing of solid-state batteries.

Here we show the potential for “Li-free” solid-state batteries using LLZO electrolytes. We demonstrate that commercially relevant capacities (≥3 mAh cm^−2^) of Li metal, comparable to current state-of-the-art Li-ion electrodes, can be both plated and stripped from an LLZO/CC interface. Based on the electrochemical results and plating behavior of the Li metal, a simple model involving interfacial forces is proposed for coupling the mechanics at the interface with the electrochemical nucleation behavior. Moreover, the work of adhesion between the CC and the LLZO is measured to enable correlation between overpotential and nucleation energy. This mechanistic insight enables the demonstration of the holy grail anode, pure, in situ-formed Li without reliance on interlayers. Finally, the performance of electrodeposited Li as a Li-metal anode is evaluated in a symmetric cell, as well as when coupled with a state-of-the-art cathode material. The results presented motivate further understanding of material interactions at solid–solid interfaces but also demonstrate the feasibility of manufacturing “Li-free” all-solid-state batteries.

## Results and discussion

### Demonstration of in situ plating of Li

**Fig. 1:**
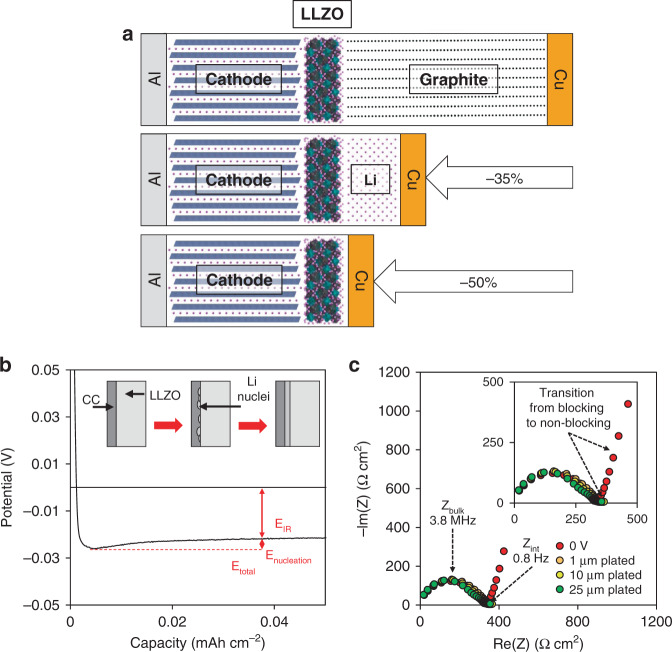
Schematic of a “Li-free” cell configuration and analysis of electrodeposition of Li onto a current collector at a constant current of 0.05 mA cm^−2^ at 25 °C. **a** Schematic of a discharged “Li-free” configuration in comparison to state-of-the-art Li-ion and a solid-state Li-metal battery with pre-deposited Li metal. Here, the current collector material is assumed to be Cu. **b** The potential response upon the initial application of a constant cathodic current, plating Li metal onto a Ni current collector. **c** The impedance spectra at several points upon the Li plating process.

Figure 1 shows the typical electrochemical response of a “Li-free” cell from open circuit voltage (OCV) through electrodeposition. The OCV begins at ∼1.8 V and then upon application of a constant current density of 0.05 mA cm^−2^, the potential quickly drops to 0 V followed by a negative potential, which indicates the onset of sustained electrodeposition of Li. After the initial drop in potential below 0 V, the potential reaches a minimum value of ∼25 mV within the first few minutes (∼3 μAh cm^−2^) of plating before asymptotically approaching a fixed steady-state value of ∼20 mV within the first hour of deposition; Motoyama et al.^20^ and Krauskopf et al.^19^ also observed the potential passing through a minimum. It has been hypothesized that this behavior is representative of the overpotential required to nucleate Li onto the CC^19,20^. Based on the electrochemical impedance spectroscopy (EIS) analysis shown in Fig. 1c, the total ionic impedance of the cell is ∼350 Ω cm^2^, which corresponds to a total DC polarization of ∼17.5 mV at a 0.05 mA cm^−2^ current density, which is reasonably consistent with the steady-state plating potential. Hence, both the galvanostatic results and the EIS spectra indicate a resistance dominated by that of the LLZO pellet after the onset of nucleation. However, as seen in Fig. 1b, the magnitude of the nucleation overpotential is significantly (∼10×) smaller than the values reported by Motoyama et al.^20^ and Krauskopf et al.^19^, which is discussed in later sections. It was also suggested by Lee et al.^23^ that the reduction in nucleation overpotential caused by a Ag-C interlayer enabled stable deposition of Li onto a CC; however, the nucleation and electrochemical behavior in alloying interlayers is not well understood. After overcoming the nucleation overpotential, the potential is relatively constant for the duration of the ∼100 h of plating. It is demonstrated in Fig. 1b that ∼5 mAh cm^−2^ of Li, corresponding to ∼25 μm of plated Li (assuming uniform deposition), can be plated without changes in the potential response. Identical tests were performed on CCs of different metals with similar results, demonstrating this capability regardless of interface chemistry. To further study the stability and kinetics of the Li/LLZO interface during in situ plating, EIS was periodically performed as Li was plated and is also shown in Fig. 1c. Initially, before any Li has been plated, the impedance spectrum exhibits capacitive behavior at low frequencies (<10 Hz) due to the blocking nature of the Ni CC to Li^+^. After Li deposition, the low-frequency regime is dominated by charge transfer, due to the non-blocking nature of Li. This is indicated by the semi-circular feature at low frequencies (0.5–5 Hz). Based on the diameter of the semi-circle, the interfacial resistance of the electrodeposited Li against the LLZO surface is <10 Ω cm^2^, which is in good agreement with previous results in which a conventional Li anode was used (e.g., foil)^18,24,25^. Furthermore, it can be seen that after the Li is initially electrodeposited, there is no change in the impedance spectra after 5 mAh cm^−2^ of Li has been plated. This confirms that no Li filaments have nucleated in the LLZO. The value of 5 mAh cm^−2^ was chosen as a relevant amount of Li commensurate with the expected areal loading of advanced cathodes. However, based on these results it is likely that significantly more Li can be plated without degradation of the LLZO at these current densities. Identical electrodeposition experiments were conducted at increasingly high current densities, but it was found that short-circuiting from Li filament propagation was consistently observed at current densities of 0.09 mA cm^−2^ and above. It is interesting to note that this value is ~10× lower than reported values of the critical current density (CCD) in symmetric Li/LLZO/Li cells^18^. This may support recent hypotheses, which suggest that the combination of inhomogeneous Li plating and slow diffusivity, and deformation of electroplated Li, can result in regions of high pressure and act as nucleation sites for Li filaments^18,19,26,27^.

**Fig. 2:**
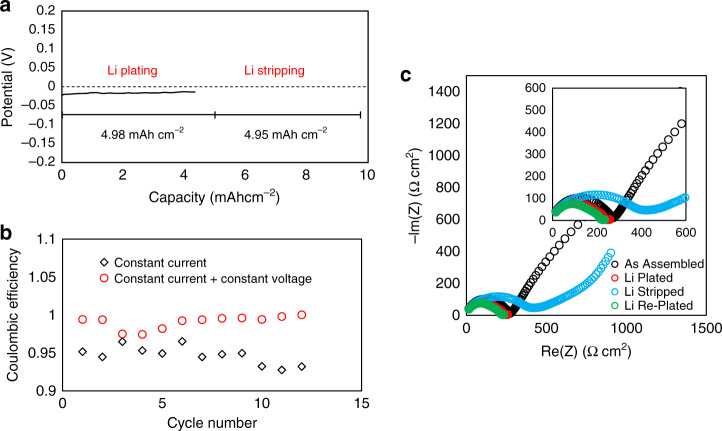
Electrochemical behavior upon Li plating and stripping at 25 °C. **a** Potential response upon plating 5 mAh cm^−2^ of Li onto a Cu current collector at 0.05 mA cm^−2^ and then subsequently stripping at 0.05 mA cm^−2^. **b** The Coulombic efficiency of Li plating and stripping of 3 mAh cm^−2^ of Li as a function of cycle number. The potential profiles are shown in Supplementary Fig. S1. **c** A comparison of the impedance spectra before and after the first Li plating and stripping cycle.

It is demonstrated in Fig. 2 that not only is it possible to electrodeposit Li at the solid–solid interface but it is also reversible. This aspect is vital, as the extent of reversibility is directly linked with the need for excess Li capacity from the cathode, if any. Similar to the potential response upon electrodeposition, a relatively constant potential is maintained upon steady-state Li stripping with the magnitude of the potential matching that upon Li plating. As the electrodeposited Li is nearly depleted, the potential dramatically increases, which is a combination of the increase in resistance associated with contact loss and the change in the OCV when transforming from a symmetric Li/Li cell back to a Li/Ni cell. Based on the amount of Li that was originally plated, it is shown that ∼99% of the Li can be returned to the source, which includes a 2 h constant voltage hold at 1.8 V. Figure 2b shows the Coulombic efficiency of Li stripping as a function of cycle number. Each cycle, the Li is plated at 0.05 mA cm^−2^, whereas the Li is stripped first at 0.05 mA cm^−2^ for three cycles, then at 0.1, 0.2, and 0.3 mA cm^−2^, each for three cycles. It can be seen that the efficiency is relatively stable above 99% over the 12 cycles. Unlike in liquid electrolytes where the loss in Coulombic efficiency is due to passivation and formation of “dead Li,” the 1% irretrievable Li observed in these experiments is more likely due to contact loss with either the LLZO or the CC. The impedance spectra before and after Li plating and stripping are shown in Fig. 2c. As demonstrated in Fig. 1, initially, the low-frequency regime (representing the Li-LLZO impedance: Z_Li-LLZO_) undergoes a transition from blocking to non-blocking behavior after the onset of Li electrodeposition. After stripping of the electrodeposited Li, the Z_Li-LLZO_ instead transitions from a non-blocking behavior to blocking behavior. Furthermore, the high frequency portion of the spectra (representing the LLZO ohmic impedance: Z_LLZO_) significantly increases, which can be attributed to the expected high degree of contact loss that occurs after removal of the interposed Li. After Li is re-plated on the subsequent cycle, not only does the low-frequency behavior return to non-blocking behavior, but the Z_LLZO_ also decreases and returns to its original value and shape. This is due to the electrodeposition of new Li, which fills the gap left by the electroplated Li from the previous cycle, therefore restoring the original contact and contact area between LLZO, CC, and Li.

### Mechanics and nucleation of Li at the CC/LLZO interface

**Fig. 3:**
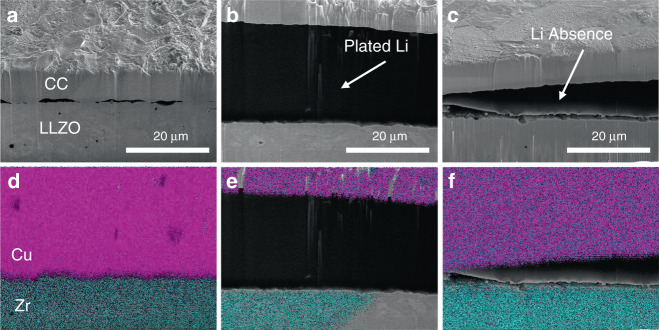
Cross-sectional SEM at the interface of the LLZO and the current collector. SEM (**a**) as assembled, (**b**) after plating 5 mAh cm^−2^ of Li, and (**c**) after plating and then stripping of 5 mAh cm^−2^ of Li. Elemental maps for Cu and Zr at the interface (**d**) as assembled, (**e**) after plating, and (**f**) after plating and stripping. Metallic Li is observed under secondary electrons in between the Cu and LLZO layer in **b** but cannot be detected since the characteristic X-ray energy falls outside of the detection range of EDS.

Figure 3 shows cross-sectional scanning electron microscope (SEM) images of the cell assembly. The pristine cell (Fig. 3a) shows minimal gaps between the Cu CC and LLZO, which is replaced by the plated Li in Fig. 3b. Figure 3b depicts the interface after 5 mAh cm^−2^ of Li has been plated, which shows the appearance of an intermediate phase, which can be identified as elemental Li, as it is unidentifiable under energy dispersive x-ray spectroscopy (EDS). Assuming uniform Li deposition, 5 mAh cm^−2^ would correspond to ∼25 μm of plated Li, but the observed layer is 33 μm, suggesting some non-uniformity in the Li deposition. Finally, after stripping of the 5 mAh cm^−2^ of Li, the intermediate phase disappears (Fig. 3c) and is replace with a 5–10 μm gap, which is consistent with the increased impedance due to contact lost shown in Fig. 2c. It should be noted that the observed gap in Fig. 3c is likely less prominent under application of stack pressure and enough contact is maintained in other areas of the interface (Supplementary Fig. 2) to maintain electrical contact.

**Fig. 4:**
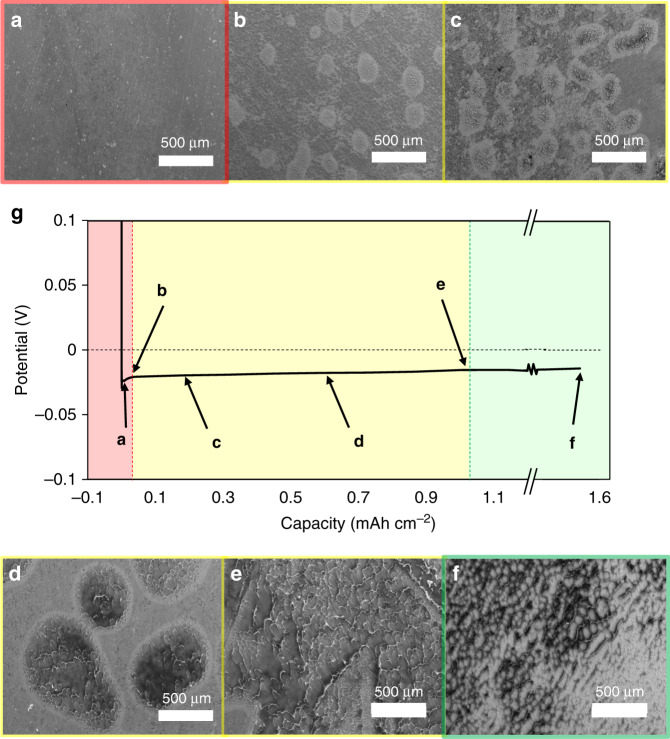
Ex situ SEM of the LLZO surface after removal of the current collector. LLZO surface after plating **a** 4 μAh cm^−2^, **b** 30 μAh cm^−2^, **c** 200 μAh cm^−2^, **d** 620 μAh cm^−2^, **e** 1.0 mAh cm^−2^, and **f** 1.6 mAh cm^−2^ of Li. The SEM images are correlated to a typical potential profile (**g**) upon plating, showing three distinct regimes: (1) Li nucleation, (2) lateral growth of the Li nuclei, and (3) coalescence of the nuclei and subsequent vertical growth of the coalesced Li film.

The presence of metallic Li can also be confirmed visually after peeling the CC off the surface after electroplating. Ex situ SEM was conducted on the LLZO surface after removal of the CC as a function of plated capacity. The morphology of the surface after deposition of 4, 30, 200, 620, 1000, and 1600 μAh cm^−2^ of Li is shown in Fig. 4. It has been previously demonstrated that at these low interfacial resistances, strong adhesion between the Li metal and LLZO is expected^25^. Therefore, while the peeling of the CC is expected to damage the Li metal itself (evidenced by the cup-cone fracture morphologies), the spatial distribution, the positions, and the dimensions of the plated Li are expected to be preserved. Although it is difficult to identify Li growths after 4 μAh cm^−2^ (5 min) of plating, after 30 μAh cm^−2^ clear patches (∼100 μm diameter) appear that cover ∼15% of the LLZO surface. Smaller Li structures are also observed which may indicate nucleation sites that did not subsequently grow. After 200 μAh cm^−2^ of Li is plated, the Li patches are observed to both increase in number and grow laterally until they begin to coalesce into a relatively uniform film after ∼1000 μAh cm^−2^. After coalescence into a uniform layer, the Li seems to grow vertically rather than laterally, which results in more height and topography of the Li film after plating 1.6 mAh cm^−2^. Overall, the SEM images clearly suggest that within the first 30 μAh cm^−2^ (38 min) of plating, some but not all of the LLZO surface has Li deposition. It should also be noted that apparently pristine LLZO interface remains after ∼12 h of plating (Fig. 4d), indicating that some regions of the LLZO surface are more resistive (perhaps due to surface chemistry), some regions of the LLZO/CC interface have a higher work of adhesion and hence are harder to open, or the LLZO/CC interface could be opened at locations adjacent to Li deposits, forming gaps that cut off current flow until nearby Li is deformed or further plated in the planar direction. After 12 h and ∼50% coverage of Li, the average height of deposited Li would be ∼6 μm lending plausibility to gap formation (i.e., 6 μm is a substantial separation distance compared to the expected surface roughness of the LLZO/CC interface). On the other hand, the cells are under 4 MPa of pressure, which may cause Li to plastically flow into portions of the CC-LLZO interface as electrodeposition occurs.

**Fig. 5:**
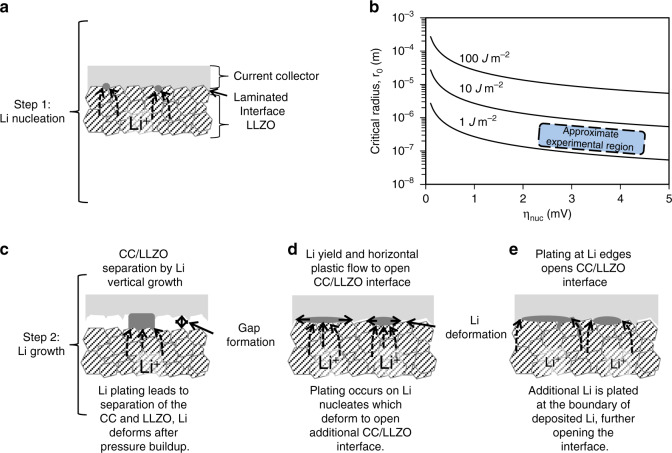
Schematic of nucleation and growth process. **a** The layers present and Li nucleates (treated as spherical in the nucleation model) and **b** the critical nucleate radius as a function of nucleation overpotential. **c**–**e** Three possible mechanisms for nucleate growth, including **c** deposition on the nucleate leading to vertical growth and separation of the adjacent CC/LLZO interface, **d** continued deposition at the nucleate center, with mechanical forces at the interfaces and Li’s low yield strength leading to horizontal plastic flow of Li, or **e** deposition at the nucleate edges.

Figure 5 envisions how the nucleation and growth process may occur. Plating likely begins with small nucleates (Fig. 5a), while subsequent growth may occur by vertical growth of Li columns (Fig. 5c), plating at the nucleate sites with horizontal plastic flow of Li to open new CC/LLZO interface (Fig. 5d, Li has a yield strength of <1 MPa and hence flows under relatively low stresses, although its plastic deformation under compression depends on boundary conditions^28^), or direct plating of Li into additional CC/LLZO interface (Fig. 5e). Both Li nucleation and growth require the delamination of the CC-LLZO interface and the formation of new Li-LLZO and Li-CC interfaces. The work associated with this process (in J m^−2^) can be estimated from the adhesion work (also in J m^−2^) of the individual contacts:

1 $$\begin{array}{*{20}{c}} {W_{\mathrm{{CC \cdot Li \cdot LLZO}}} = W_{\mathrm{{adh,CC \cdot LLZO}}} - W_{{\mathrm{{adh,}}}{\mathrm{Li \cdot CC}}} - W_{{\mathrm{{adh}}}\,{\mathrm{Li \cdot LLZO}}}} \end{array}$$

This equation assumes that new Li-LLZO and Li-CC interfaces completely fill the opened CC-LLZO interface. Also note that the Li plating work and interfacial energies in Eq. (1) include all of the mechanisms that can contribute to the adhesion of two interfaces^29^. Peel tests were conducted to experimentally estimate the works of adhesion of the CC on the LLZO, which is described in the Supplementary Information (Supplementary Fig. 4). The experimental measurements of *W*_adh, CC·LLZO_ provide estimates (Supplementary Methods) on the order of 1–10 J m^−2^ (for Cu and Ni), which is higher than surface energy estimates, which only consider the bonding energy at atomically smooth interfaces^24^. Reducing this value may be of practical importance in optimizing anode-free cell manufacturing. To provide a quantitative framework for step (1) (the creation of the first Li nucleates) the critical radius (i.e., the radius at which continued growth is favored thermodynamically) for a spherical nucleate of Li can be determined using the Gibbs free energy of formation of the nucleate:

2 $$\begin{array}{*{20}{c}} {{\Delta}G_{\mathrm{{total}}} = - \frac{4}{3}\pi r^3{\Delta}G_{\mathrm{{Li}}} + 4\pi r^2W_{\mathrm{{CC \cdot Li \cdot LLZO}}}} \end{array}$$

Here, Δ*G*_*Li*_ is the energy associated with Li deposition, and *W*_CC Li·LLZO_ is the work associated with opening the CC-LLZO interface, as given by Eq. (1). Here, Δ*G*_Li_ is for the reaction *Li*^+^ + *e*^−^ → *Li* and we can also use Δ*G*_Li_ = −*Fη*_nuc_ where *F* is Faraday’s constant and *η*_nuc_ is the Li nucleation overpotential. The critical radius—below which no Li should nucleate—can be determined by:

3 $$\begin{array}{*{20}{c}} {r_0 = - \frac{{2W_{\mathrm{{CC \cdot Li \cdot LLZO}}}\bar V_{\mathrm{{Li}}}}}{{F\eta _{\mathrm{{nuc}}}}}} \end{array}$$

$\bar V_{Li}$ is the molar volume of Li. Figure 5b illustrates the critical radius as a function of *η*_nuc_ for several values of *W*_CC·Li·LLZO_. Here, the value of *η*_nuc_ can be thought of as the additional potential that needs to be supplied to do the work required to first open the CC-LLZO interface. As shown in Fig. 5b, assuming a value of ∼1 J m^−2^, and assuming that both *W*_adh,Li·CC_ and *W*_adh,Li·LLZO_ are negligible, critical radii are in the micron range for several mV of overpotential. The assumption that *W*_adh,Li·CC_ and *W*_adh,Li·LLZO_ are negligible is justified if the adhesion mechanisms for the diffusion-bonded interface (∼1–10 J m^−2^) are much stronger than the adhesion mechanisms for the new interfaces of CC and LLZO with Li metal (which, if similar to calculated surface energies, are <1 J m^−2^)^24^. In other words, 10 s of J m^−2^ of work to open the diffusion bonded CC/LLZO interface corresponds to several mV of potential driving force to create the first Li nucleates, and the potential profile at short times in Fig. 1b may reflect the nucleation process.

During step (2) (the subsequent growth of Li nucleates), work is required to continue opening the CC-LLZO interface, with possible mechanisms for that opening shown in shown in Fig. 5c–e. For any of these mechanisms, the overpotential required to open the CC-LLZO interface is given by:

4 $$\begin{array}{*{20}{c}} {\eta _{\mathrm{{CC \cdot Li \cdot LLZO}}} = \left( {W_{\mathrm{{CC \cdot Li \cdot LLZO}}}\frac{{dA}}{{dt}}} \right)/I} \end{array}$$

*I* (in A) is the applied current and *dA*/*dt* (in m^2^ s^−1^) is the rate of CC/LLZO interface opening. The SEM images in Fig. 4 suggest the rate of opening of the CC/LLZO interface (i.e., *dA*/*dt*)) may be nonlinear, such than the overpotential for interface opening during Li growth varies with time. The overpotential described in Eq. (4) may also contribute to the potential profile seen in Figs. 1 and 4. We also note that if the nucleate is subjected to an applied pressure, that would further increase the potential driving force required to drive the nucleation and growth process. An order of magnitude estimate for the additional potential is given by $\eta \approx \frac{{\bar V_{Li}}}{F}p$, where *p* is the pressure in the nucleate. An applied pressure of 4 MPa therefore results in an additional *η* ≈ 0.5 mV. Future research can clarify which mechanisms shown in Fig. 5c–e are most important and how they depend on boundary conditions such as applied pressure and current density.

An obvious limitation of this analysis is that it neglects the nm-scale processes associated with Li plating, including at polycrystalline LLZO grains of various orientations and with surface roughness. The results shown in Fig. 5 should therefore be considered as an initial framework for how the relatively high work of adhesion, measured in this study, may influence the overpotentials for nucleation and growth. It should also be emphasized that the observations made in the SEM analysis—that a small number (Fig. 4 shows ∼10 nucleates per mm^−2^) of nucleates appear to grow large (Fig. 4b shows a diameter of ∼100 µm by 1 h) rather than many small nucleates forming and quickly merging – is a strong indication of surface heterogeneity. However, we acknowledge that although present experiments have not identified which surface heterogeneity(ies) (e.g., in *W*_CC·Li·LLZO_, interfacial LLZO kinetics, CC surface properties, etc.) are most important. The proposed model applies to the initial Li plating cycle into the pristine diffusion-bonded CC-LLZO interface, subsequent stripping and plating cycles require further analysis.

Motoyama et al.^20^ has also measured and proposed one of the first models for Li plating into a CC/solid-electrolyte interface, modeling the potential evolution at the start of plating in a thin-film battery, with a LiPON solid electrolyte and a pulsed laser deposited (PLD) or sputter deposited metal CC with a thickness of 30 to 90 nm or 1 μm. These authors could optically observe, for the thinner CCs for which Li beneath them was easily visible, lithium nucleation and growth and, in some cases, fracture of the lithium through the CC. They observed a voltage rise of ∼30 mV (for the 90 nm CC) to ∼50 mV (for the 30 nm CC), while for the 1 µm CC the voltage rise was, as in our experiments, <10 mV. They developed a hoop stress model for pressure buildup in the lithium nucleates to account for the potential profile, assuming a fixed 0.2% strain in the region of CC adjacent to the nucleate, and setting the hoop radius to fit the data. Their model does not account for work of adhesion or interfacial energies between the phases, including during the growth of lithium nucleates, and requires the interfaces to be in full contact as GPa-level pressures are generated within the Li nucleate. The mechanical properties are assumed to be those of the bulk metals, which may not be the case, especially for the <100 nm PLD-deposited metal films. In addition, their model does not address why Li nucleates form and grow with tens of mV of overpotential while substantial areas of the CC receive no Li plating. In our view, this fact is clear evidence of the need to consider more closely spatial variations in interfacial resistance, and to identify the contributing mechanisms, for Li nucleation into a CC/solid-electrolyte interface.

### Performance of an in situ-plated Li-metal anode

**Fig. 6:**
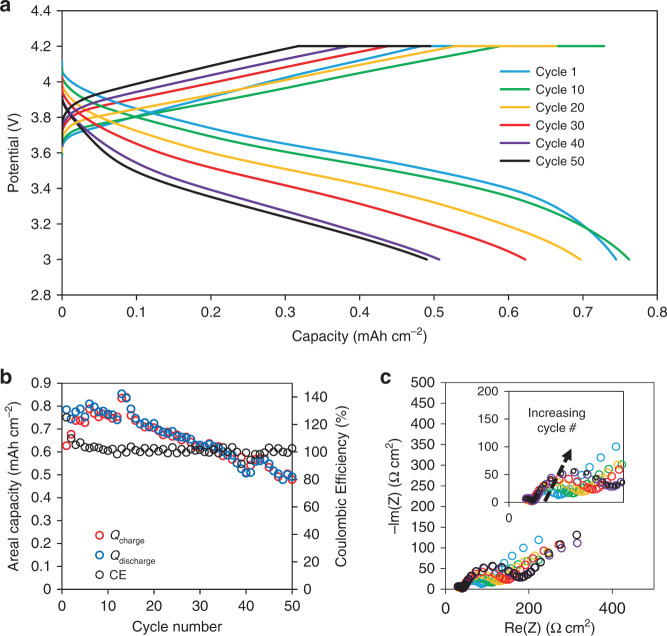
Cycling behavior of a Li-metal battery composed of an NCA/PEO-composite cathode, LLZO, and in situ-plated Li-metal anode cycling at 60 °C. **a** Representative charge/discharge curves cycling at a C/10 rate. **b** The areal capacity and Coulombic efficiency as a function of cycle number. **c** The impedance spectra measured after every ten cycles.

The results presented thus far have demonstrated the capability of electrochemically forming thick Li-metal films at the interface of the LLZO electrolyte and CC. To evaluate the performance of the in situ formed Li-metal anode with a relevant model cathode, a Li-metal/LLZO/NCA all-solid-state cell was fabricated. To avoid the need for small quantities of liquid electrolyte to improve the NCA/LLZO interface charge transfer kinetics, an all-solid-state PEO/NCA composite cathode was used, as it has been demonstrated that the PEO/LLZO interface resistance can be reduced to relatively low values (<200 Ω cm^2^ at room temperature)^30^. However, as the bulk conductivity of polyethylene oxide/lithium bis(triflouromethanesulfonyl)imide (PEO-LiTFSI) polymers is low at room temperature (∼1 × 10^−6^ S cm^−1^), the cell was operated at 60 °C. After assembly of the Cu CC, LLZO, and PEO/NCA cathode, the cell undergoes a single formation cycle, which plates the Li-metal anode at 0.05 mA cm^−2^ from the Li contained within the NCA. Of the available 3 mAh cm^−2^ cathode capacity, 2.7 mAh cm^−2^ (∼13.5 μm) was plated upon formation and used as the Li-metal anode. Figure 6 shows the charge/discharge behavior, cycling at 60 °C (±3 °C) at a C/10 rate (0.27 mA cm^−2^), following the formation cycle. Prior to full cell testing, the CCD was measured (Supplementary Fig. 5) in a symmetric cell at room temperature. It was observed that the CCD at room temperature of a 25 μm in situ formed Li anode (0.3 mA cm^−2^) was notably lower than reported values using Li foil (∼1 mA cm^−2^), which may suggest the presence of length-scale-dependent phenomena that impact Li filament initiation. It is seen in Fig. 6a the cell undergoes a typical potential response upon charging and discharging and achieves a capacity of 0.75 mAh cm^−2^ on the first cycle. Although some first-cycle capacity loss is expected for NCA following the formation cycle, it is believed that the lack of an optimized PEO-composite cathode prevents the majority of the capacity from being accessed at a C/10 rate. A cell was also cycled at 80 °C at the same current density as the formation cycle (0.05 mA cm^−2^), and it was shown that significantly higher capacities (∼2.4 mAh cm^−2^), closer to the capacity of the formation cycle, can be achieved (Supplementary Fig. 6). Figure 6b shows the capacity over 50 cycles along with the Coulombic efficiency. Coulombic efficiencies above 100% are observed which is not impossible in this case, as less Li is cycled (<0.8 mAh cm^−2^) than what was originally plated (2.7 mAh cm^−2^) and therefore for any given discharge cycle, there may be more accessible Li than what was previously plated upon charging. Moreover, as LLZO is stable against Li, the absence of side reactions should, in principle, enable 100% Coulombic efficiency, a paradigm shift in Li battery operation. Despite a few outlying cycles, the Coulombic efficiencies remain near 100% throughout the 50 cycles. Even with the high efficiencies, a ∼25% capacity fade is observed after 50 cycles, which can be attributed to the increase in cell impedance. Figure 6c shows an increase in the total cell impedance from ∼90 Ω cm^2^ on the 1st cycle to ∼200 Ω cm^2^ on the 50th cycle. As the change is isolated to low frequencies, the impedance growth is solely due to changes at an internal interface. As the capacity of the in situ-formed Li anode is known to be 2.7 mAh cm^−2^ based on the formation cycle, and <0.8 mAh cm^−2^ is being cycled, the anode/electrolyte interface is that of excess Li/LLZO, which was seen in the earlier results to be unchanging under these depths of discharges. Therefore, the cause is more likely related to cycling-induced degradation and increasing charge transfer resistance of the PEO/LLZO or PEO/NCA interface, rather than degradation of the Li/LLZO interface. Although the oxidative stability of solid-polymer electrolytes is dependent on a number of factors like salt concentration and chemical composition, it is possible that degradation of the PEO above ∼3.8–4.0 V may be the cause of the impedance growth and subsequent capacity fade observed in Fig. 6 and Supplementary Fig. S6^31–36^. To confirm this, a Cu/LLZO/Li-foil cell was cycled under identical conditions and no increase in cell impedance nor capacity or Coulombic efficiency fade was observed (Supplementary Fig. 7). This strongly suggests that the observed capacity fade is limited by the cathode/catholyte interface rather than the in situ-plated Li anode, further highlighting the need for continued development of stable, high-rate, and compatible composite cathodes.

“Li-free” manufacturing enabled through in situ-plated Li-metal anodes could dramatically impact the feasibility of solid-state batteries for vehicle electrification. The ability to electrochemically form the Li-metal anode not only eliminates the processing costs and challenges of working with bulk or vapor-deposited Li metal, but may also enable the usage of commercially available Li-ion cathode materials without the volumetric and gravimetric penalty of having pre-deposited Li. In this work, it has been demonstrated that significant capacities (5 mAh cm^−2^) of Li metal can be both electrodeposited and depleted at the interface of LLZO and a CC without any LLZO degradation, without the need for an interlayer, and with high efficiencies. Based on the morphology and distribution of plated Li as a function of plated capacity, a simple model based on interfacial forces was proposed to describe the nucleation and lateral growth of isolated Li regions into a coalesced uniform film. Finally, a prototypical all-solid-state battery was fabricated by in situ plating the Li from a PEO/NCA composite cathode, through the LLZO and onto a Cu CC. The Li/LLZO/PEO-NCA cell was cycled for 50 cycles at a C/10 rate, exhibiting capacities of 0.8 mAh cm^−2^ upon the first few cycles and near 100% Coulombic efficiencies. Although capacity fade was observed after 50 cycles, the lack of efficiency fade suggests that the cell is limited by the composite cathode performance, providing further motivation for solid-state battery cathode development. The results presented here not only demonstrate the first “Li-free” solid-state battery based on garnet electrolytes, but also highlights the need for further investigation of how mechanics, electrochemistry, and spatial variations in interfacial properties affect Li electrodeposition at solid–solid interfaces.

## Methods

### Cell assembly

In this study, CC/LLZO/Li cells were fabricated using commercial metal foils, hot-pressed LLZO pellets, and Li foil. Li-metal foil was used as the Li source primarily because it has been demonstrated in several other works that Li/LLZO interfaces can be reliably and consistently fabricated with low interfacial resistances without any intermediate coatings or modifications^24,25,37,38^. In comparison, there have not yet been many demonstrations of facile charge transport across cathode/LLZO interfaces which may induce artifacts in the nucleation behavior at the CC/LLZO interface. LLZO pellets of the composition Li_6.5_La_3_Zr_1.5_Ta_0.5_O_12_ were synthesized by a solid-state synthesis method and then consolidated by rapid-induction hot-pressing as described by Taylor et al.^39^ and cut into pellets ∼2 mm-thick on a diamond saw. Battery grade Ni (35 μm) and Cu (10 μm) foils were purchased from Targray and used as CCs. To attach the CCs to the LLZO surface, one side of the LLZO was polished with 1200 grit sandpaper and then the CC was placed onto that surface. The CC was then laminated to the LLZO by applying a pressure of 3–6 MPa at temperatures between 900–1100 °C, depending on the CC metal, for 5 min. After laminating, the opposite LLZO face was then polished using a variety of sandpapers and diamond pastes to a final polish of 0.1 μm. As previously described, the CC/LLZO was then heat-treated at 400 °C in Ar to remove resistive surface layers and then 750 μm Li foil (Alfa Aesar) was pressed onto the polished surface at elevated temperatures^18^.

Full cells were fabricated by first laminating a Cu CC (10 μm) to the LLZO pellet and heat-treating at 700 °C in Ar. A composite cathode was composed of a PEO-LiTFSI catholyte and a commercially cast state-of-the-art NCA electrode for conventional Li-ion batteries (University of Michigan Battery Lab). The NCA electrode was cast with an active loading of ∼3 mAh cm^−2^ and an approximate porosity of 35%. The PEO-LiTFSI catholyte was synthesized as described by Gupta et al.^30^ and then infiltrated into the cathode porosity by heating the PEO and uniaxially pressing into the cathode against the heat-treated LLZO surface under a pressure of 4.2 MPa and held for several hours at a temperature of 80 °C.

### Electrochemical methods

Electrochemical experiments were performed using a Bio-logic VMP-300 galvanostat/potentiostat. After fabricating the CC/LLZO/Li cells, Li was plated onto the CC by applying a constant current such that Li^+^ was transported from the Li-metal source toward the CC. Low current densities in the range of 0.05−0.1 mA cm^−2^ were used for in situ plating under a stack pressure of ∼4 MPa. A temperature of 25 °C is used for all cycling tests. EIS was used to monitor the state-of-health of the cell and to confirm the presence of Li metal at the CC/LLZO interface. EIS was conducted using a 5 mV perturbation voltage at frequencies between 500 mHz and 7 MHz. Li stripping experiments were performed by applying a constant current density in the range of 0.05−0.3 mA cm^−2^ with the opposite polarity, such that Li^+^ was transported away from the CC/LLZO interface toward the Li-foil source.

After assembly of the Cu/LLZO/PEO-NCA full cells, the Li-metal anode was formed by plating the Li from the NCA onto the Cu at a constant 0.05 mA cm^−2^ at 80 °C. After formation of the Li-metal anode, the cells were cycled at 60 °C with a constant current, constant voltage scheme at a C/10 rate (0.27 mA cm^−2^) between 4.2 V and 3.0 V, with a 1 h constant voltage hold at 4.2 V. EIS was performed after every tenth charge cycle with a 5 mV perturbation voltage between 500 and 7 MHz.

### Materials characterization

In order to visualize CC/LLZO interfaces, cross-sections were cut using focused ion beam (FIB) milling, imaged, and analyzed under EDS using a Thermo Fisher Helios G4 Plasma FIB UXe. Surface analysis was done using a Hitachi S3500N scanning electron microscope. Just prior to evacuation of the SEM chamber, the CC was peeled off the LLZO surface to reveal the plated Li. Although there is some exposure to air, the removal of the CC was done as quickly as possible to minimize the exposure.

## Supplementary information

Supplementary Information

## Data Availability

The data that support the findings of this study are available from the corresponding author upon reasonable request.
