# Publisher Correction: Enabling “lithium-free” manufacturing of pure lithium metal solid-state batteries through in situ plating

**DOI:** 10.1038/s41467-020-20374-y

**Published:** 2020-12-10

**Authors:** Michael J. Wang, Eric Carmona, Arushi Gupta, Paul Albertus, Jeff Sakamoto

**Affiliations:** 1 Department of Materials Science and Engineering, University of Michigan, Ann Arbor, MI 48109 USA; 2 Department of Chemical and Biomolecular Engineering, University of Maryland, College Park, MD 20742 USA; 3 Department of Macromolecular Science and Engineering, University of Michigan, Ann Arbor, MI 48109 USA; 4 Department of Mechanical Engineering, University of Michigan, Ann Arbor, MI 48109 USA

**Keywords:** Batteries, Batteries, Nanoscale materials

Correction to: *Nature Communications* 10.1038/s41467-020-19004-4, published online 15 October 2020.

Figure 2a contains an error where the signal for Li stripping is missing in the published version. This is now corrected.

