# Supplementary material for: Enabling “lithium-free” manufacturing of pure lithium metal solid-state batteries through in situ plating
Source: Nat Commun. 2020 Oct 15;11:5201. doi: 10.1038/s41467-020-19004-4 (PMC7567811; doi:10.1038/s41467-020-19004-4)
Supplement: Supplementary file 1 — Supplementary Information [file 41467_2020_19004_MOESM1_ESM.pdf]

Supplementary Information

Enabling "lithium-free" manufacturing of pure lithium metal solid-state batteries through *in situ* plating

Wang *et al.*

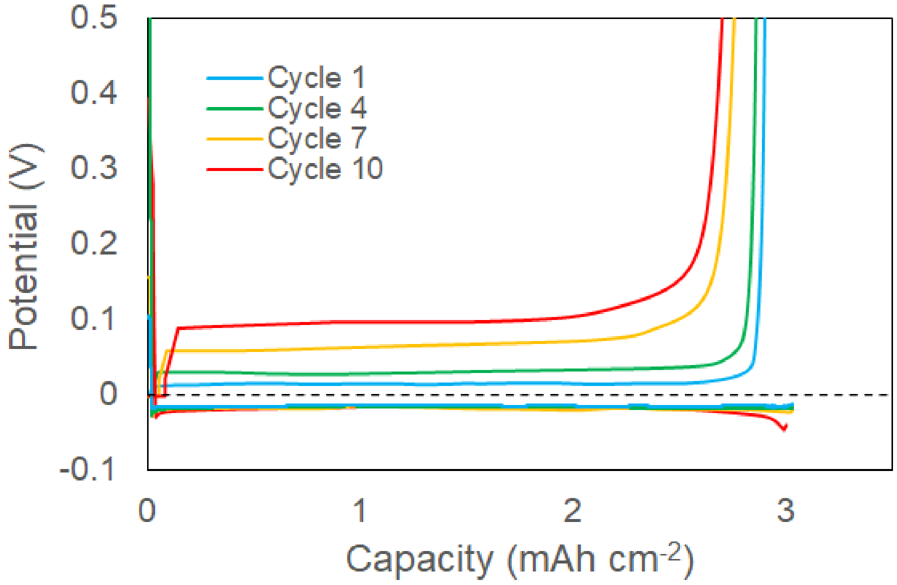

Supplementary Figure 1 | Potential profiles over several plating and stripping cycles at different current densities corresponding to the data plotted in Figure 2b.

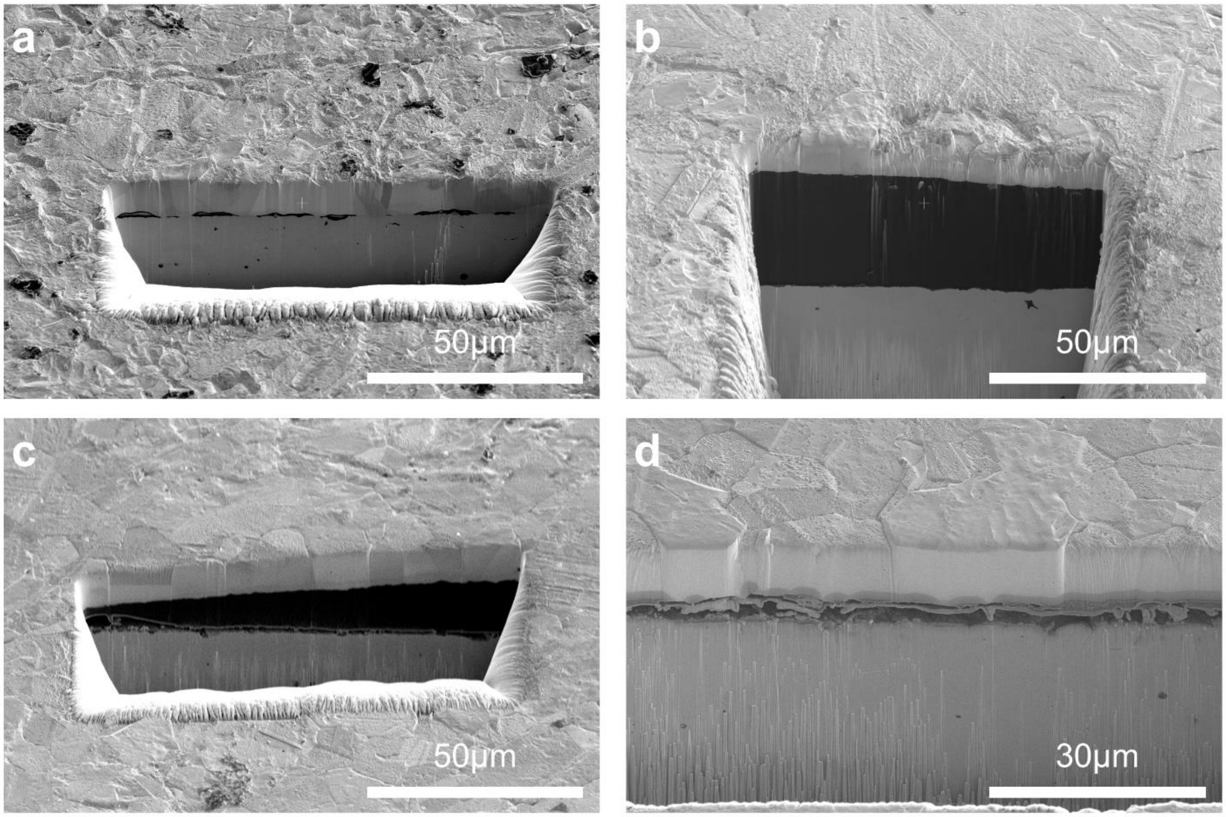

Supplementary Figure 2 | Low magnification SEM of FIB-milled Cross-Sections from Figure 3. (**a**) Cu current collector as laminated onto LLZO, (**b**) after 5 mAh cm^{-2} of Li is plated, and (**c**) after 5 mAh cm^{-2} of Li is plated and stripped. The intermediate Li layer in between the Cu and the LLZO in (**b**) more clearly shows textural features at lower magnifications which indicates the presence of an intermediate phase rather than empty space, despite the high color contrast. Not all regions of the interface after stripping 5 mAh cm^{-2} of the plated Li exhibit such a prominent separation between Cu and LLZO that is observed in (**c**). One such region of a less pronounced separation is shown in (**d**), which exhibits a smaller gap between Cu and LLZO but is still more pronounced than in (**a**) and shows a similar residue between Cu and LLZO that is also observed in (**c**).

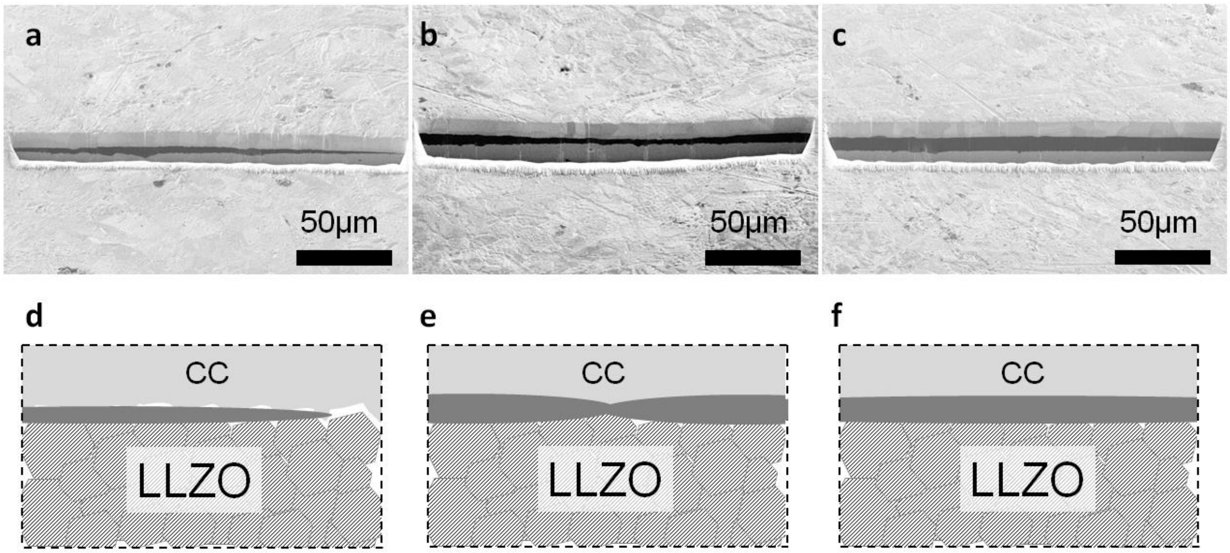

Supplementary Figure 3 | SEM of FIB-milled cross-sections for different capacities of Li plated onto a Cu current collector. (**a**) 200 µAh cm^{-2} Li plated, (**b**) 620 µAh cm^{-2} Li plated, and (**c**) 1.0 mAh cm^{-2} Li plated. The capacities plated correspond to Figure 4c-e. Schematics illustrating the progression from (**d**) isolated Li patches, (**e**) coalescence of the Li patches, and (**f**) formation of a uniform Li layer.

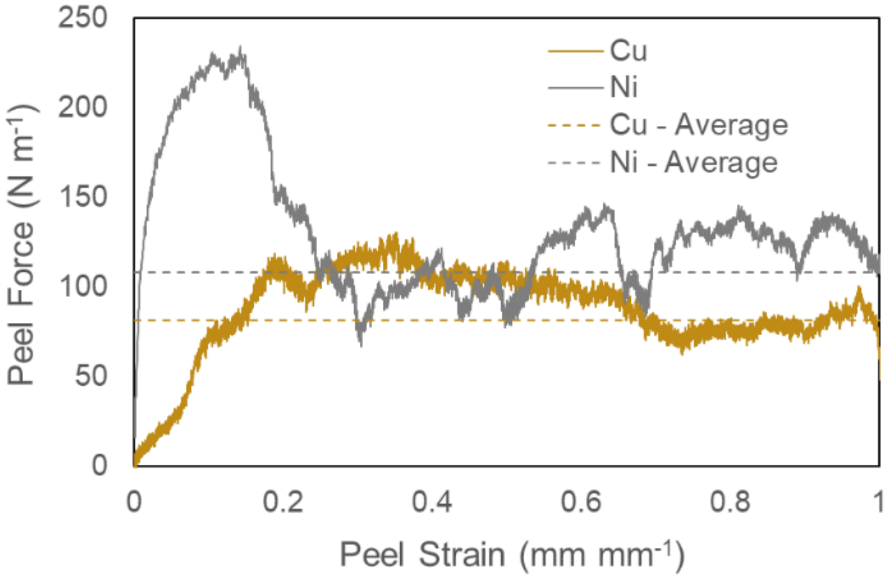

Supplementary Figure 4 | Peel Tests for Measuring Macro-scale Work of Adhesion. 90° Peel tests to measure the work of adhesion of the laminated current collectors on the LLZO surface. The dashed lines indicate the average peel force for each current collector type for three different samples each. The average peel force is measured from the average force over the steady-state region (~0.4 – 1.0 peel strains). The average work of adhesions for Cu and Ni are estimated to be within the range of 1-10 J m^{-2} as described in the Supplementary Methods section.

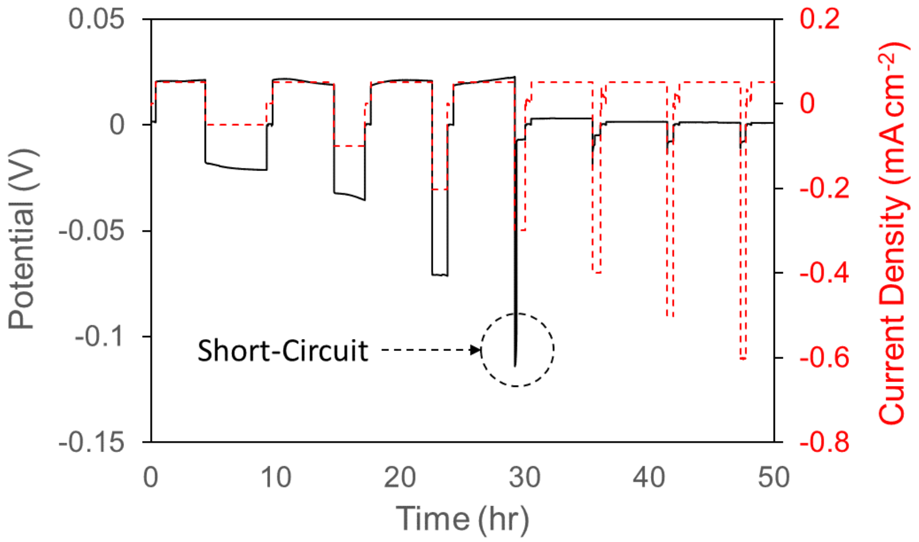

Supplementary Figure 5 | Critical Current Density Measurement of 25 µm *in situ* Plated Li

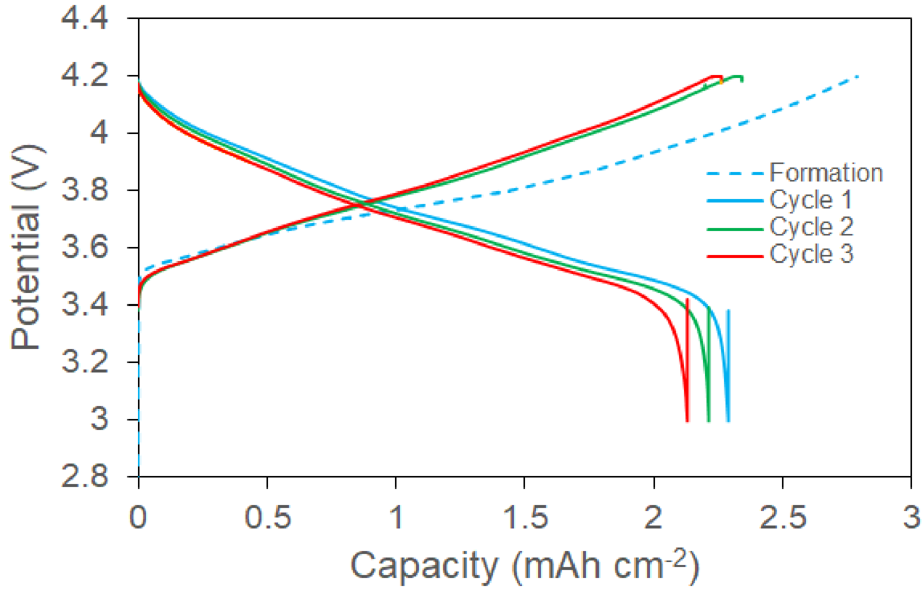

Supplementary Figure 6 | Cycling behavior of a Li metal battery composed of an NCA/PEO composite cathode, LLZO, and *in situ* plated Li metal anode cycling at 80°C.

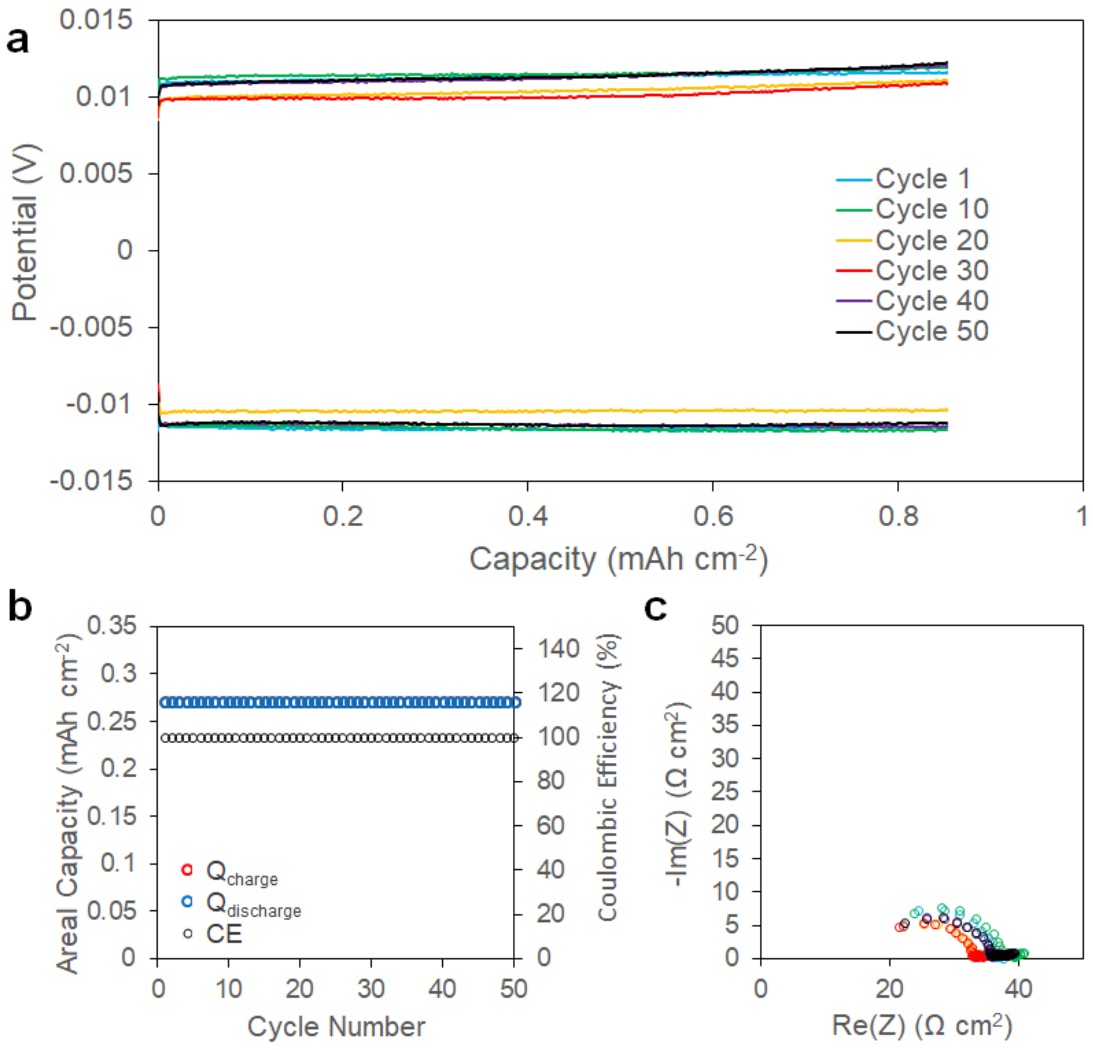

Supplementary Figure 7 | Cycling behavior of a Li metal battery composed of a Li metal foil, LLZO, and *in situ* plated Li metal anode cycling under identical conditions as in Figure 6. The *in situ* anode is formed with a capacity of 2.7 mAh cm^{-2} with 0.85 mAh cm^{-2} (corresponding to the maximum capacity value in Figure 6b) being cycled at 60°C . (**a**) Representative charge/discharge curves cycling at a C/10 rate. (**b**) The areal capacity and Coulombic efficiency as a function of cycle number. (**c**) The impedance spectra measured after every ten cycles.

Supplementary Methods

Peel Tests

To measure the work of adhesion of the laminated current collectors on the LLZO surface, peel tests were conducted using a TA.XTplus Textra Analyzer with a 90° Degree Peel Rig. Peel tests were conducted based on a modified version of the ASTM D6862 - 11(2016) method[1]. Ni and Cu foils cut to a width of 4 mm and length of 12 mm were laminated onto the LLZO under the conditions described in the Experimental Methods Section. Peel tests were conducted at a rate of 10 mm min^{-1} until complete delamination of the current collector. The work of adhesion could be estimated based on the following equation[2–5]:

$$\Gamma = P(1 - cos\varphi) - Q \qquad (Supplementary\ Equation\ 1)$$

Where P is the average peel force per width, ϕ is the peel angle, Q is the work required to deform the current collector, and Γ is the work of adhesion. The peel force is measured from the steady state regime, after the initial transient region and before fracture of the interface. Although a 90° peel rig was used, the actual peel angles were measured to be 45° for the Ni current collectors and 81° for the Cu current collectors. While an exact value for Q is difficult to obtain due to its dependence on several variables, based on ideal models[3–5], its value can be estimated by the following relationship:

$$Q = \frac{\sigma_Y^2 t}{2E}\left(2\sqrt{\frac{6EP}{\sigma_Y^2 t}(1-\cos(\varphi'))} - 5 + \frac{10}{3\sqrt{\frac{6EP}{\sigma_Y^2 t}(1-\cos(\varphi'))}}\right) \qquad (Supplementary\ Equation\ 2)$$

Where σ_Y, t, and E are the yield strength, thickness, and elastic modulus of the current collector, and φ′ is the effective peel angle accounting for curvature near the interface. In this case it is assumed that the effective peel angle and the measured peel angle are identical. It should be noted that due to the

ideality of the model along with other forces that may exist at the CC/LLZO interface, the estimated work of adhesions represents an upper bound of the true values.

Supplementary Discussion

Discussion of critical current density differences in Supplementary Figure 5

The critical current density was measured by applying a current density of 0.05 mA cm^{-2}, passing a total charge of 0.25 mAh cm^{-2}, and then reversing the direction of the current and passing an equal amount of charge in the opposite direction. After each cycle, the current density is increased. In order to avoid Li filament initiation at the interface of the LLZO and the Li source foil, the current density was only increased when plating onto the *in situ* plated Li. After first electrochemically forming a 25 µm thick (5 mAh cm^{-2}) Li metal anode, the CCD was measured by cycling 0.25 mAh cm^{-2} (1 µm) of Li with increasing current densities. The amount of charge passed in each cycle was chosen to be comparable to other reports in the literature. In this particular measurement, the current density is maintained constant while plating onto the Li source, in order to ensure that failure occurs upon the charging of the in situ plated Li metal anode. The dramatic drop in cell potential at 0.3 mA cm^{-2} is indicative of the formation and propagation of Li filaments, which result in an internal short-circuit. The CCD of 0.3 mA cm^{-2} of the in situ plated Li anode is particularly low in comparison to other reports of the CCD of LLZO. Under nearly identical measurement conditions, the CCD of symmetric Li/LLZO cells constructed with >100 um thick Li electrodes is typically measured to be ~1 mA cm^{-2}. It is unclear why the CCD is notably lower in the case of the *in situ* plated Li compared to bulk Li, although two possibilities are of note. First, the surface roughness of the CC/LLZO interface is rougher than the typical Li/LLZO interface as was previously reported. The rougher surface may concentrate either stress or ionic flux, leading to a more premature fracture of the electrolyte. Secondly, the aspect ratio of the 25 µm thick Li is significantly larger than the aspect ratio of typical >100 µm thick foils. As it has been demonstrated that the mechanical properties of Li metal are highly dependent on the length scale[9–11], the difference in aspect ratio may lead to different mechanical behavior of the *in situ* plated Li compared to bulk Li. However, future work is needed to better understand the CCD behavior of thin (< 20 µm) *in situ* formed Li anodes.

Supplementary References

1. D14 Committee. *Test Method for 90 Degree Peel Resistance of Adhesives*. http://www.astm.org/cgi-bin/resolver.cgi?D6862-11R16 doi:10.1520/D6862-11R16.
2. Wei, Y. & Hutchinson, J. W. Interface strength, work of adhesion and plasticity in the peel test. in *Recent Advances in Fracture Mechanics* (eds. Knauss, W. G. & Schapery, R. A.) 315–333 (Springer Netherlands, 1998). doi:10.1007/978-94-017-2854-6_16.
3. Kim, J., Kim, K. S. & Kim, Y. H. Mechanical effects in peel adhesion test. *Journal of Adhesion Science and Technology* **3**, 175–187 (1989).
4. Simlissi, E., Martiny, M., Mercier, S., Bahi, S. & Bodin, L. Elastic–plastic analysis of the peel test for ductile thin film presenting a saturation of the yield stress. *Int J Fract* **220**, 1–16 (2019).
5. Aravas, N., Kim, K.-S. & Loukis, M. J. On the mechanics of adhesion testing of flexible films. *Materials Science and Engineering: A* **107**, 159–168 (1989).
6. Xu, C., Ahmad, Z., Aryanfar, A., Viswanathan, V. & Greer, J. R. Enhanced strength and temperature dependence of mechanical properties of Li at small scales and its implications for Li metal anodes. *Proceedings of the National Academy of Sciences* **114**, 57–61 (2017).
7. Herbert, E. G., Dudney, N. J., Rochow, M., Thole, V. & Hackney, S. A. On the mechanisms of stress relaxation and intensification at the lithium/solid-state electrolyte interface. *J. Mater. Res.* **34**, 3593–3616 (2019).
8. Fincher, C. D., Ojeda, D., Zhang, Y., Pharr, G. M. & Pharr, M. Mechanical Properties of Metallic Lithium: from Nano to Bulk Scales. *Acta Materialia* S1359645419308754 (2019) doi:10.1016/j.actamat.2019.12.036.
